# ST Elevations and Ventricular Tachycardia Secondary to Coronary Vasospasm upon Extubation

**DOI:** 10.1155/2020/1527345

**Published:** 2020-02-12

**Authors:** Patrick J. Lindsay, Rachel C. Frank, Edward A. Bittner, Sheri Berg, Marvin G. Chang

**Affiliations:** ^1^Department of Anesthesia Critical Care and Pain, Massachusetts General Hospital, Boston, Massachusetts, USA; ^2^Cardiology Division, Massachusetts General Hospital, Boston, Massachusetts, USA

## Abstract

ST elevations (STE) in the perioperative setting can result from a number of different etiologies, the most common and feared being acute coronary syndrome (ACS). However, other causes should be considered, as treatment may differ depending on the diagnosis. Here, we describe a case of STE and ventricular tachycardia in a patient at high risk for ACS. The patient had a prior diagnosis of coronary vasospasm; however, given pre-existing risk factors, much consideration and deliberation occurred prior to electing conservative therapy. This report provides an overview of perioperative vasospasm and other causes of STE, which anesthesiologists should be aware of.

## 1. Introduction

Coronary vasospasm, also known as Prinzmetal angina, has been reported in patients receiving general anesthesia and regional anesthesia [1]. This may result in myocardial ischemia and ultimately hemodynamic collapse [2]. Suspected triggers include catecholamine release, vasoconstrictor medications, redistribution of blood flow, and increase in blood pH [3].

Coronary vasospasm usually manifests with severe chest pain, mimicking acute coronary syndrome. However, this is often not evident in an anesthetized patient. Although the most common cause of ST elevations (STE) in the perioperative setting is acute coronary syndrome (ACS), alternative diagnoses should be considered to ensure the patient does not undergo unnecessary and invasive investigations [4]. ST-changes in coronary vasospasm usually last less than 15 minutes, with the electrocardiogram (ECG) spontaneously returning to baseline [5].

In this case, we describe a patient with a previous diagnosis of coronary vasospasm, with multiple risk factors for acute coronary syndrome, who developed ST elevations and wide complex tachycardia. We review the differential diagnosis of STE and criteria for distinguishing between the different diagnoses. Written consent for publication was provided by the patient.

## 2. Case

A 60-year-old transgender woman presented for bilateral orchiectomy for gender reassignment surgery. Her past medical history included non-Hodgkins lymphoma treated with chemotherapy, Hodgkins lymphoma treated with radiation, hypertension, and dyslipidemia. She had previously undergone bariatric surgery in 2005, with no perioperative complications. All preoperative laboratory work was within normal limits.

Since 2010, the patient has experienced multiple episodes of angina which were investigated with myocardial perfusion imaging tests (MIBI), transthoracic echocardiogram (TTE), and coronary angiograms, all of which were negative for evidence of myocardial ischemia. The most recent MIBI and TTE were 2 months prior to surgery, both of which were unremarkable. Salient preoperative cardiovascular medications included isosorbide mononitrate, aspirin, and metoprolol, which ultimately helped to improve her chest pain.

On preoperative evaluation, the patient reported good exercise tolerance, with no chest pain or shortness of breath with exertion. Her physical examination was unremarkable with a BMI of 32.6 and normal vital signs. She was deemed appropriate for surgery and underwent general anesthesia. There was no hemodynamic instability during the case, and no ECG changes were noted prior to extubation. Blood loss during the case was minimal, and she appeared well resuscitated. At the end of the case, she was breathing spontaneously without support at a rate of 17 and was extubated. Moments later, a number of ectopic beats were identified on the ECG tracing, with transient ST-segment changes. These changes were new in comparison with her baseline; therefore, a 5-lead ECG was displayed on the monitor. Leads II, III, and aVF demonstrated profound STE (>3 mm), with leads V5 and aVL having clear ST-depressions and T-wave inversions. Approximately 30 seconds after STE were noted, and the patient's ECG progressed into a wide complex ventricular tachycardia, lasting between 15 and 30 beats, before returning to ST elevations in leads II, III, and aVF and ST-depressions in reciprocal leads. The patient transitioned between STE and wide complex tachycardia on four separate occasions, lasting a total of 6 minutes. Throughout this event, radial pulse was palpable; however, no arterial line was in place. Therefore, it remains unclear if hypotension occurred, although noninvasive blood pressure taken every minute during this period of time, measuring a systolic blood pressure not lower than 105 mmHg. Due to residual effects of the anesthesia, the patient was unable to verbalize whether she had chest pain during the episode. Of note, during these episodes of wide complex tachycardia, the patient was completely still eliminating the possibility of artifact contributing to the ECG tracing. Unfortunately, no ECGs were obtained from the operating room, as the cardiac arrest cart was being mobilized, the cardiology team was being called, and the patient's stability was prioritized. The monitor alarm history was reviewed; however, the ST-changes that were observed on the 5-lead ECG during the event were not recorded fully, only 3 lead interpretations were available.

As the patient had a number of risk factors for ACS, the cardiology team was called to the OR to assess the patient for cardiac catheterization. By the time the STEMI team arrived, the ECG changes had resolved. A bedside transthoracic echocardiogram (TTE) was performed after STE and tachycardia resolved, which revealed normal biventricular function and no significant wall motion abnormalities. Blood work was sent immediately to assess for electrolyte abnormalities, and with all laboratory values remaining within normal limits compared to the preoperative period.

Given the self-limited STE and recent preoperative stress test being negative, the diagnosis of coronary vasospasm was made. The case had been planned to be ambulatory surgery; however, she was admitted for monitoring overnight with telemetry. Home medications (isosorbide mononitrate and metoprolol) were continued with isosorbide mononitrate changed to twice daily dosing. She remained asymptomatic during the admission without further ECG changes or arrhythmia and was discharged the following day.

## 3. Discussion

**Figure 1:**
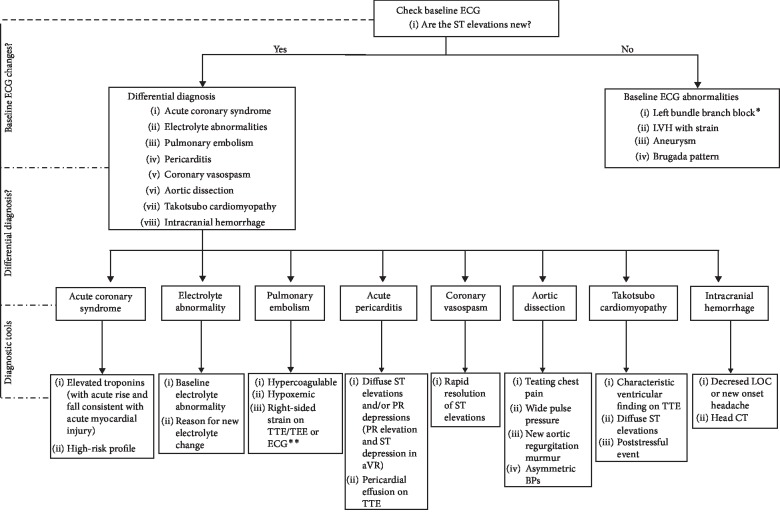
Approach to perioperative ST elevations: ^*∗*^LBBB may be intermittent or rate related. ^*∗∗*^Evidence or right heart strain on ECG includes new right axis deviation, right bundle branch block, S wave in lead I, Q wave in lead III, and T-wave inversion in lead III. The most common ECG finding with pulmonary embolism is sinus tachycardia. Other findings include T-wave inversions in V1–V4, prominent R wave in V1, clockwise rotation (shift of transition point R > S, closer to V6), and right atrial abnormality. ECG: electrocardiogram, TTE: transthoracic echocardiogram, TEE: transesophageal echocardiogram, LOC: level of consciousness, LVH: left ventricular hypertrophy, and BP: blood pressure.

Coronary vasospasm can present in a number of ways including STE, ventricular arrhythmias, asystole, and bradycardia. This may result in myocardial ischemia and possibly hemodynamic collapse [1]. When assessing STE in the perioperative setting, a number of conditions should be considered including ACS, pericarditis, Takotsubo cardiomyopathy, and coronary vasospasm.  Figure 1 demonstrates an approach to STE in the perioperative setting [6]. Careful assessment of the patient should be undertaken to ensure the correct diagnosis is made, with treatment pathways varying substantially.

The etiology of coronary vasospasm in the perioperative setting is unclear, although it is likely associated with catecholamine release, vasoconstrictor medications, redistribution of blood flow, and increase in blood pH [3]. There are limited reports of coronary vasospasm in the context of extubation in the literature. In this case, it is suspected that extubation triggered catecholamine release with subsequent vasospasm, thus leading to STE and ventricular tachycardia. Although there is a lack of data of its benefits, one might consider a deep extubation for patients at high risk of coronary vasospasm in an attempt to prevent the sympathetic drive that can be associated with extubation.

Treatment of coronary vasospasm includes nitroglycerin in the acute setting, with calcium channel blockers and long-acting nitrates used as long-term treatments [7]. In this circumstance, the spontaneous resolution of ECG changes and hemodynamic stability in the postoperative setting warranted no nitroglycerin acutely. Additionally, she had neither further arrhythmias nor ST-changes during her admission. Given she remained asymptomatic both prior to and following the surgery, she was discharged on the twice daily isosorbide mononitrate and metoprolol with plans to follow-up with her outpatient cardiologist and to discuss the addition of calcium channel blockers for prophylaxis. Her last coronary angiogram was in 2010, with all vessels being patent. Her outpatient cardiologist will determine whether a repeat angiogram is indicated.

From a prevention perspective, there have been a number of case reports outlining coronary vasospasm in the perioperative setting in the context of abrupt cessation of vasodilators [8–10]. Although our patient continued her nitrates up until the surgery, she still experienced suspected coronary vasospasm. The severity and duration of her vasospasm may have been attenuated given that she had continued her nitrates preoperatively.

A number of factors made this case challenging. Firstly, the patient had a number of risk factors for coronary artery disease including sex, age, hypertension, dyslipidemia, BMI, exogenous hormone use, and previous thoracic radiation for lymphoma. Secondly, coronary artery spasm is suspected to be associated with ischemic heart disease, and the patient had been suffering from coronary vasospasm for many years [11]. Finally, the stress of surgery also placed the patient higher risk for cardiac events [12]. As a result, careful decision making was required to determine whether a coronary angiogram was necessary given the patient's risk factors and clinical setting. After review of her prior extensive diagnostic testing, it was deemed that this case of STE and wide complex tachycardia was likely secondary to acute vasospasm. This was supported by the rapid resolution of the STE, without residual ischemic changes on the ECG or wall motion abnormalities on bedside TTE [5, 13]. Therefore, both the anesthesiology and cardiology teams determined that coronary vasospasm was most likely and felt comfortable in not pursuing further diagnostic testing unless symptoms reoccurred. It is important to note that this decision should not be taken lightly, given the clinical consequences of untreated acute coronary syndrome. This case highlights coronary vasospasm as a cause of both persistent and self-limited ST-elevation ECG changes aside from ACS that perioperative providers should be aware of.
